# Experimental warming of a mountain tundra increases soil CO_2_ effluxes and enhances CH_4_ and N_2_O uptake at Changbai Mountain, China

**DOI:** 10.1038/srep21108

**Published:** 2016-02-16

**Authors:** Yumei Zhou, Frank Hagedorn, Chunliang Zhou, Xiaojie Jiang, Xiuxiu Wang, Mai-He Li

**Affiliations:** 1 Shanghai Institute of Technology, Shanghai 201418, China; 2 Swiss Federal Research Institute WSL, Zuercherstrasse 111, 8903 Birmensdorf, Switzerland; 3 Changbai Mountain Forest Ecosystem Research Station, Chinese Academy of Sciences, Baihe 133613, China; 4 Institute of Applied Ecology, Chinese Academy of Sciences, Shenyang 110016, China

## Abstract

Climatic warming is expected to particularly alter greenhouse gas (GHG) emissions from soils in cold ecosystems such as tundra. We used 1 m^2^ open-top chambers (OTCs) during three growing seasons to examine how warming (+0.8–1.2 °C) affects the fluxes of carbon dioxide (CO_2_), methane (CH_4_) and nitrous oxide (N_2_O) from alpine tundra soils. Results showed that OTC warming increased soil CO_2_ efflux by 141% in the first growing season and by 45% in the second and third growing season. The mean CH_4_ flux of the three growing seasons was −27.6 and −16.7 μg CH_4_-C m^−2^h^−1^ in the warmed and control treatment, respectively. Fluxes of N_2_O switched between net uptake and emission. Warming didn’t significantly affect N_2_O emission during the first and the second growing season, but stimulated N_2_O uptake in the third growing season. The global warming potential of GHG was clearly dominated by soil CO_2_ effluxes (>99%) and was increased by the OTC warming. In conclusion, soil temperature is the main controlling factor for soil respiration in this tundra. Climate warming will lead to higher soil CO_2_ emissions but also to an enhanced CH_4_ uptake with an overall increase of the global warming potential for tundra.

High-latitude and altitude tundra ecosystems are generally considered as one of the world’s most sensitive areas in response to global warming^1^. On the one hand, climatic warming of the earth’s surface is expected to be particularly strong at high northern latitudes and at high elevations^2^,^3^. On the other hand, soils of ‘cold’ ecosystems store large stocks of labile soil carbon^4^ and the temperature sensitivity of biogeochemical processes is the highest in cold climates^5^, suggesting that the greenhouse gas balance of cold systems responds particularly sensitive to the ongoing climate warming. Hydrological changes may lead to tradeoff between carbon dioxide (CO_2_) and methane (CH_4_) balance of ecosystems underlain by permafrost, with exchange rate of CO_2_ being usually several orders of magnitude greater than that of nitrous oxide (N_2_O) and CH_4_. However, CH_4_ and N_2_O have a greater warming potential than CO_2_. Hence, CH_4_ and N_2_O fluxes might be equally important to the greenhouse gas balance as soil CO_2_ effluxes^6^,^7^.

The net exchange rates of CH_4_, CO_2_ and N_2_O between soil and atmosphere are determined by the balance between sources and sinks. The fluxes of these greenhouse gases (GHG) are highly complex involving a number of soil biochemical processes, the composition of soil microbial and plant communities, as well as abiotic conditions^8^. Soil CO_2_ emission are mainly driven by temperature, moisture, and the supply with substrate from plants^5^,^9^,^10^. Climate warming is generally increasing soil CO_2_ effluxes, but the overall net C balance of soils depends also on the C inputs by the vegetation^11^,^12^. For tundra and cold treeline ecosystems, it has been found that respiratory carbon losses responded more sensitive to increases in temperature than carbon gains by plants, which turned tundra ecosystems from a carbon sink into a carbon source^13^,^14^,^15^. Soil heating by 2.5 °C in a wet tundra increased soil CO_2_ emission by 39% at northeast coast of Greenland^1^. However, responses of soil respiration to warming are often short-lived and ecosystem-dependent^16^,^17^.

Fluxes of CH_4_ were found to be highly sensitive to climate warming in various cold ecosystems. Upland boreal soils in Siberia were found to consume atmospheric CH_4_ particularly during dry and warm summer months^18^. Warming is potentially increasing CH_4_ uptake due to soil drying and greater CH_4_ diffusion into soils^7^. In contrast, soil warming could decrease CH_4_ release by increasing soil NH_4_^+^ contents through a stimulated N mineralization which suppress the activities of methanotrophic bacteria^19^,^20^. In contrast, wetlands release large amounts of CH_4_ because methanogenesis is induced by anaerobic soil conditions. For instance, measurements in a high-arctic wet tundra ecosystem showed that the mean growing season CH_4_ fluxes ranged between 2.94 and 4.60 mg m^−2^ h^−1^, and were positively correlated with soil temperature^21^. However, in a boreal forest in interior Alaska, Wickland *et al.*^22^ did observe only weak relationships between CH_4_ flux and temperature. At an alpine treeline, Karbin *et al.*^20^ found that soil warming induced a shift in the depth distribution of methanotrophic bacteria, which, however, was not associated with significant responses of CH_4_ fluxes.

The N_2_O exchange between soil and atmosphere plays an important role in climate warming, global nitrogen cycling and stratospheric ozone depletion^23^. Climate warming may increase N_2_O fluxes by an accelerated N cycling but it may decrease the fluxes from partly anaerobic soils by a declining soil moisture^23^,^24^,^25^. Experimental warming (+3.5 °C) increased N_2_O emission in upland grassland where mean annual temperature was 8.7 °C^23^. However, the warming effects were only observed during the growing season but not in winter.

The majority of warming experiments in tundra quantifying fluxes of greenhouse gases have been carried out in arctic region^26^,^27^. The aim of our study was to investigate the responses of GHG fluxes to warming in a mountain tundra of China. In China, tundra exists only in Altai Mountain and Changbai Mountain. Changbai Mountain is a dormant volcano reaching up to 2734 m a.s.l. where the tundra zone is above 2000 m a.s.l.^28^. At the Songjiang weather station near Changbai Mountain, mean annual temperature in the region increased at a rate of 0.6 °C per decade from 2.2 °C in 1978 to 3.9 °C in 2006^29^. In our study, here, we established open-top chambers (OTCs) according to the ITEX (International Tundra Experiment) standard to increase the growing season temperature (June to September). We have measured GHG fluxes in response to OTC-warming for three growing seasons from 2011 to 2013. Generally warming will lead to lower soil moisture. Thus, we expected that growing season warming will increase greenhouse gas exchange due to accelerated microbial activities and to a lower soil moisture promoting the gas diffusion into soils. Consequently, we hypothesized that the warming during the growing season will increase soil CO_2_ effluxes, but also the uptake of CH_4_ and N_2_O.

## Result

### Climatic variables

Mean air temperature during the growing seasons was 0.8 to 1.2 °C higher in the OTCs than in the control plots during the whole study period. The increase in mean soil temperature at 10 cm depths was about 0.2 °C. OTCs had negligible effects on relative air humidity, averaging +1.1%. Soil water contents were 0.02 to 0.03 m^3^m^−3^ higher in the OTCs as compared to the control plots during the three growing seasons, which corresponded to an increase of approximately 10%.

### Soil CO_2_ efflux

**Figure 1:**
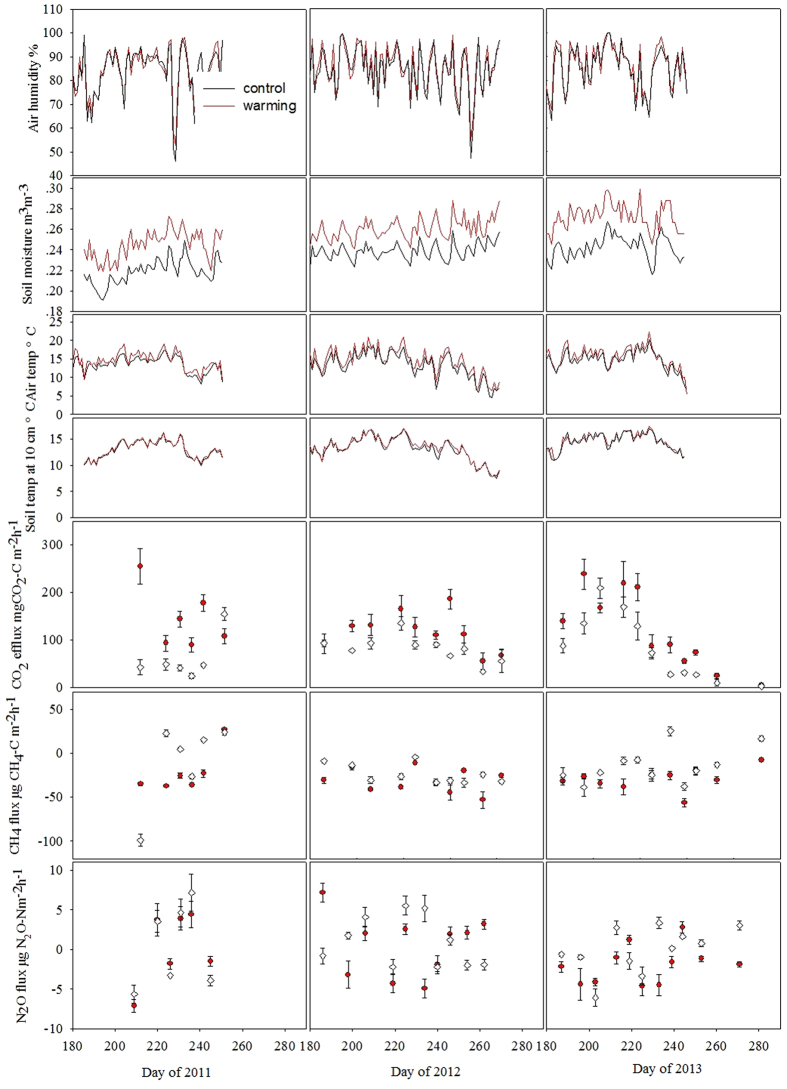
Air relatively humidity, soil moisture, air temperature, soil temperature at 10 cm depth; CO_2,_ CH_4_ and N_2_O fluxes for the warming OTCs (red) and the control plots (black) over three growing seasons’ period from 2011 to 2013. Data show mean ± SE.

Warming increased soil CO_2_ efflux across three growing seasons (*P* = 0.017), but the magnitude of effect decreased from +141% in the first treatment year to approximately +45% in the following two years (Table 1). The mixed effect model for individual years indicated significant warming effects in 2011 and 2013, but not in 2012. The relatively high effect of OTC warming on soil CO_2_ efflux during the 2011 growing season was mainly attributed to the pulse in July (Fig. 1), but treatment effects were still significant without the initial peak (*P* = 0.035). Averaged across the three growing seasons in the Changbai Mountain tundra, the respiratory CO_2_ loss was 61% higher from the OTC warming than from the control plots.

**Figure 2:**
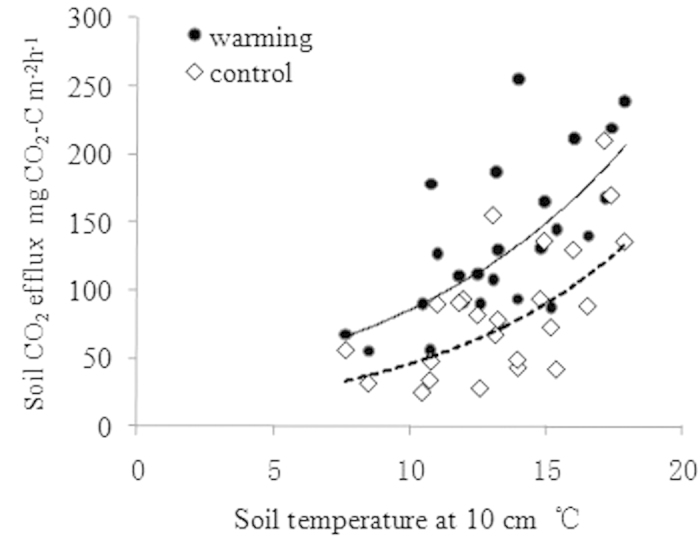
The relationship between soil temperature at 10 cm depth and soil CO_2_ efflux. The Q_10_ values were 3.0 for the warming OTC (circles) and 3.8 for the control plot (diamonds), respectively.

At a given soil temperature, soil CO_2_ effluxes were higher in the OTCs than in the controls and soil CO_2_ efflux increased exponentially with soil temperature at 10 cm depth for both OTCs and control plots (Fig. 2). The corresponding Q_10_ value in the control plots was 27% higher than that in the OTCs. No significant relationships between CO_2_ effluxes and air or soil moisture were found because of little variation of moisture throughout the experimental period (Fig. 1).

### CH_4_ Flux

Tundra soils in Changbai Mountain were a net sink for CH_4_ with OTC warming increasing the sink strength (Fig. 1; Table 1). Net production of CH_4_ was observed on 6 out of 27 measurements in the control treatment, but only at one date under OTC warming. Over the three-year long experimental period, OTC warming significantly increased CH_4_ flux (*P* = 0.014), but this effect differed among dates (*P*_Treatment×Time_ < 0.001). In 2011 and 2013, there were significantly higher CH_4_ flux from the OTC warming than from the control plots (+122 and +96%, respectively; *P* < 0.05), but not in 2012 (+32%; *P* = 0.121). Averaged among all measurements, soils were a net sink for CH_4_ with −27.6 μg CH_4_-C m^−2^h^−1^ in the warmed and −16.7 μg CH_4_-C m^−2^h^−1^ in the control treatment. Over the entire measurement period and all plots, there were no significant relationships between CH_4_ fluxes and soil moisture as well as temperatures in air and soil.

### N_2_O flux

In all years and in both treatments, N_2_O fluxes in Changbai mountain tundra switched between positive and negative values. Overall, the soils were a net N_2_O source during the growing seasons in 2011 and 2012, but a net N_2_O sink in 2013 (*P*_season_ = 0.042). The experimental warming using OTCs tended to affect N_2_O fluxes (*P* = 0.094, across all three years). There was a significant difference between the two treatments for a number of measurements (12 out of 27 dates). However, as the direction of the treatment effect was not consistent, the warming effect during whole growing seasons were not significant in 2011 and 2012, but significant in 2013 when soils were mostly a N_2_O sink (*P* = 0.042; Table 1). In the third treatment year, OTC warming increased the mean N_2_O uptake during the growing season from −0.08 μg NO_2_-N m^−2^h^−1^ in the control plots to −1.95 μg NO_2_-N m^−2^h^−1^ in the OTC plots (*P* = 0.003). We did not find obvious relationship between N_2_O fluxes and microclimatic parameters either on an annual basis or for individual measurements.

### Global warming potential

The GWP considering the fluxes of all three gases was dominated by soil CO_2_ effluxes (Table 1). Including CH_4_ and N_2_O fluxes decreased the GWP of CO_2_ emissions by less than 1%. Over the entire three growing seasons, OTC warming increased the GWP significantly by 70% (*P* = 0.018).

## Discussion

Greenhouse gas fluxes from cold ecosystems such as tundra are assumed to respond particularly sensitive to climatic warming^18^,^30^,^31^,^32^. Our experimental warming in the Chinese Changbai Mountain tundra using OTCs supports this assumption. The passive warming increased soil CO_2_ effluxes and CH_4_ uptake for three growing seasons which was consistent with our hypothesis. The likely reasons for the altered greenhouse gases fluxes from the mountain tundra are an increased microbial activity and a change in the soil microclimate.

The stimulated belowground activity under warming is indicated by an accelerated soil respiration, which is in agreement with a number of warming studies in alpine and arctic environments^17^,^33^,^34^. Concurrent measurements of soil microbial biomass in the Changbai Mountain tundra did not reveal a significant warming effect in 2012^35^, suggesting an enhanced metabolic activity of the soil microbial community with a less efficient use of C and smaller allocation of C into microbial growth^36^. The declining C use efficiency is in line with other studies in mountain ecosystems^37^,^38^ which can be explained by differential effects of temperature on growth and respiration^36^,^39^. In our OTC study, we found that the stimulation of soil respiration decreased strongly with treatment time. This decline is in agreement with other warming experiment and it is usually attributed to an acclimation of soil CO_2_ effluxes to warming either by a substrate depletion, an adaptation of soil microbial communities or a decreasing C allocation of plant assimilates to the rhizosphere^16^,^38^,^40^,^41^,^42^. Due to the moderate warming in our OTC study with an increase in air and soil temperature by less than 1.5 °C and relatively high soil organic C contents of 7% in the uppermost 10 cm, it seems unlikely that the depletion of labile soil carbon was primarily responsible for the strong decline after first growing season. Here, however, we can only speculate about the exact mechanism, but we rather relate the rapid and short-lived initial warming effect to an altered C allocation to the rhizosphere, which responds highly sensitive to microclimatic changes even at a daily time scale^43^.

The increased soil CO_2_ effluxes do not necessarily imply increased net ecosystem C losses because a frequently observed stimulation in plant growth in tundra ecosystems by warming^12^,^17^ potentially increases C inputs and hence, counterbalances C losses. In our study, we have indeed found a mean growth stimulation of approx. 7% in aboveground vegetation height (unpublished data), suggesting at least a partial offset of the enhanced soil CO_2_ effluxes by an increased plant growth. However, here, we cannot estimate a complete C balance, but a two-decade long summer warming in the Alaskan tundra by Sistla *et al.*^12^ indicated largely unchanged total soil C pools despite increased C cycling rates.

Our results also showed that soil respiration exponentially increased with soil temperature at 10 cm depth for both OTCs and control plots. This is in agreement with a number of studies observing a close positive relationship between soil respiration and temperature^44^,^45^,^46^,^47^. Soil moisture is often reported to control soil respiration^48^,^49^. However, in our study, soil moisture did not significantly affect soil respiration for both warming OTCs and control plots, probably because soil moisture ranged always between 0.2 and 0.3 mm^−3^ and hence in an range assumed to be optimal for respiratory activity. Moreover, it did not vary strongly during the whole measurement period and between treatments. We therefore conclude that soil temperature was the main controlling factor for soil respiration in this tundra. The relationship of soil CO_2_ efflux with temperature indicated that at a given temperature, the effluxes were higher in the warmed OTCs than the control plots (Fig. 2). This pattern suggests that the warming effect in our study goes beyond a simple temperature response, which we attribute to an indirect warming effect through an enhanced plant growth and to a higher allocation of assimilates to the belowground by the warming of soil under ‘cold’ conditions^43^.

The measurement of CH_4_ fluxes indicates that Changbai Mountain tundra is a CH_4_ sink which is in agreement with other observations in cold ecosystems with ‘upland’ soils in northern Siberia and the Alps, as well as in the high arctic tundra in Canada^18^,^20^,^46^. In our study, the OTC warming increased the CH_4_ sink strength by 66% (Table1), supporting experimental warming studies in the Canadian high Arctic^32^ and in the subarctic Scandes^50^. There are two potential reasons for the increased CH_4_ uptake under warming, higher soil temperature and a change in soil moisture^51^. Higher soil temperatures could enhance CH_4_ fluxes by accelerating microbial processes and enzyme activities. However, increases in soil temperature can stimulate both methanotrophic activity and methanogen activity at the same time^52^, which may balance out CH_4_ production and consumption in warmer soils^53^. In our study, we did not observe a significant relationship between CH_4_ uptake and temperature (air and soil temperatures) when data were aggregated over three growing seasons, but we cannot rule out that for individual measurements, warmer soil temperatures have induced a higher activity of soil methanotrophs. Similar poor correlations have also been found in other ecosystems, including tundra^7^,^20^,^53^. Alternatively, the increased CH_4_ uptake could be induced by an associated decrease in soil moisture under warming, because CH_4_ fluxes are closely related to the moisture status of soils by its effects on anaerobicity and gas diffusivity^54^,^55^. Higher temperatures generally induce a decline in soil moisture and hence, soil warming could decrease methanogenesis and facilitate CH_4_ diffusion into soils^56^. However, in our study, OTC warming increased soil moisture by about 10% due to the blocking of winds. We therefore conclude that it is unlikely that soil moisture was responsible for the greater CH_4_ uptake in the Changbai Mountain tundra. In contrast, the increased soil moisture might have partly balanced out the presumably positive temperature effect on soil methanotrophs. Thus, we expect that the increase in the CH_4_ sink strength we have observed here will even be larger during the ‘natural’ ongoing climate warming with an associated decline in soil moisture.

Observed responses of N_2_O flux to warming are highly variable, ranging from enhanced uptakes to increased emissions in various ecosystems^7^, even in the same ecosystem under different vegetation types^26^. In the present study in the Changbai Mountain tundra, N_2_O fluxes also varied between net emissions and net uptakes on an inter- and intra-annual timescale. Warming treatment alleviated the N_2_O emission in 2011 and 2012 though not significant but strongly enhanced N_2_O uptake during the growing season 2013. Similar results were found by Hu *et al.*^45^ in an alpine meadow where the effects of a less than 2 °C warming during the growing season on N_2_O fluxes varied from positive to negative responses with year and season. Also, in a temperate heathland ecosystem, a plateau peatland and a treeline ecotone, elevated temperatures did not significantly affect N_2_O fluxes^20^,^51^,^57^. The likely reason for the small and inconsistent responses in our and other studies are the number of processes involved with warming potentially accelerating N-cycling and hence denitrification^58^.

## Conclusion

Our results indicated that the three-year OTC warming during growing season increased soil CO_2_ effluxes in the Changbai mountain tundra, but at the same time stimulated CH_4_ uptake and decreased N_2_O emission. Soil CO_2_ emissions dominated the global warming potential and OTC warming increased the overall GHG fluxes from soil. However, the GHG fluxes from soils might have partly been balanced out by an enhanced C uptake by plants and we thus expect the effect of warming to be smaller for the overall ecosystems GHG balance. We also have to recognize that our measurements were confined to growing seasons and for an annual ‘complete’ budget, estimates of GHG fluxes during the long winter would be needed. In addition, warming responses are frequently short-lived and only long-term manipulation experiments may represent the ongoing climatic warming. We therefore assume that our experiment is indicative for the intra-annual variability with greater GHG emissions to be expected during warmer summers.

## Methods

### Site description

The study was conducted in a tundra ecosystem at an elevation of 2028 m a.s.l., Changbai Mountain in northeastern China (41°58′–42°42′N; 127°67′–128°27′E). The climate is characterized by long and cold winters and short and cool summers. The mean annual temperature is −1.6 °C, with the highest mean diel temperature of 28 °C in August and the lowest one with −35 °C in January^59^. The distribution of precipitation over the year is uneven with the largest amount of rainfall in July and August. The study site is dominated by dwarf shrubs of *Dryas octopetala* var. *asiatica*, *Vaccinium uliginosum* and *Rhododendron aureum*, with an average coverage of 56%, 29% and 7%, respectively. Additional species are *Carex atrata*, *Polygonum ochotense* etc. The mean height of the vegetation canopy is about 8 cm during growing season (June to September). In the uppermost 10 cm of soil, the total contents of organic carbon, nitrogen and phosphorus were 6.95%, 0.37% and 0.54%, respectively.

### Experimental design

Ten hexagon open-top chambers (OTCs) were installed on tundra according to the criteria of International Tundra Experiment in June 2010. The distance between two adjacent chambers was about 4 to 5 meters. All chambers were placed on relatively flat ground surface with similar vegetation cover. The experimental area almost covered 300 m^2^. The chambers were made of translucent plexiglas which had a high solar transmittance. They were 45 cm high and had inwardly inclined sides (60 cm at bottom, total area approx. 1 m^2^) which helped to trap heat and decrease wind speed. The control plot was set adjacent to each OTC within two meters having the same area and a similar vegetation. We used two sets of HOBO (Bourne, MA, USA) weather station locating in the OTC and the adjacent outside control, at a height of 15 cm above the ground surface recording air temperature, air relative humidity and photosynthetically active radiation (PAR) every half hour. Soil temperature and moisture was measured at 10 cm depth. Due to the thick snow cover, strong winds and low temperatures during winter in Changbai tundra, the experiment could only be conducted during the growing season, which lasted on average from June until September.

### Flux measurements

Fluxes of CH_4_, CO_2_ and N_2_O fluxes were measured once a week during the growing season (June to September) from 2011 to 2013 except during rainy weather. The fluxes of the three greenhouse gas were measured using the static chamber method^60^ with six permanently installed cubic stainless-steel collars (length × width × height = 15.6 × 15.6 × 25 cm each) which were inserted into the soil to a depth of 6 cm in three OTCs and three control plots. Vegetation in the collars was removed during the whole measurements. For each measurement, a stainless-steel chamber was placed for 30 minutes on a water-filled rim of each collar providing a gas tight seal. Air temperature was measured inside each chamber with thermocouples. Gas samples were taken with a 30 cm^3^ plastic syringe four times at equal intervals of 10 min. The gas sampling was usually carried at about 9:00 a.m. at local time to represent daily mean flux, since previous studies in tropic forests and wetlands revealed that the flux at 9:00 a.m. almost equals the daily mean flux^48^,^60^. Sample gases were transported to the laboratory and were analyzed within four hours using gas chromatography (Hewlett 5890, USA). The CH_4_, CO_2_ and N_2_O flux rates were calculated from the slope of the temporal change in gas concentrations within the closed chamber. Further details of the method can be found in Song *et al.*^60^. Positive values refer to the flux from the soil to the atmosphere (emission) and negative values refer to the flux from the atmosphere to the soil (uptake or consumption).

### Data analysis and statistics

The relationship between soil respiration (*SR*) and soil temperature at 10 cm depth (*T*) was estimated by fitting the following exponential function to the data of the three growing seasons:


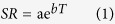


where a and b were coefficients. The Q_10_ values were then calculated with Q_10_ = e^10b^.

The net global warming potential (GWP) from soils was calculated as sum of net emissions of CO_2_, N_2_O and CH_4_ by converting each gas unit to CO_2_ equivalent at a 100-yr time scale with climate change feedbacks using a conversion factor of 1 for CO_2_, 298 for N_2_O and 34 for CH_4_. The warming effect on GWP was estimated by the difference of the GWP in the warmed and control treatment.

The effects of warming and measurement date on CH_4_, CO_2_ and N_2_O fluxes was analyzed by fitting mixed-effects models by maximum likelihood (http://www.R-project.org/). The models included the sequential fixed effects *Block*, *Treatment* (Control vs. OTC chamber), as well as *Time* and *Plot* as the random effect accounting for the split-plot design and repeated measurement structure. Sampling times were used as categorical variables. Residuals of repeated measures showed a first-order autoregressive covariate structure, which was included in the model using the corAR1 function. For each measurement, the significant difference in three gas fluxes between the warming OTCs and the control plots was also assessed by One-way ANOVA followed by a Least Significance Difference (LSD) test using SPSS 16.0 system (SPSS Inc., Chicago, IL, USA). The dependent variables were all log or square-root transformed to meet the assumptions of normality and homoscedasticity. The relationship between temperature and moisture and gas fluxes was tested for all data of the three growing seasons using Pearson’s correlation analyses. Differences at the *P* < 0.05 level were considered significant.

## Additional Information

**How to cite this article**: Zhou, Y. *et al.* Experimental warming of a mountain tundra increases soil CO_2_ effluxes and enhances CH_4_ and N_2_O uptake at Changbai Mountain, China. *Sci. Rep.* **6**, 21108; doi: 10.1038/srep21108 (2016).

## Figures and Tables

**Table 1:** The mean ( ± standard error) CO_2_, CH_4_, N_2_O fluxes and global warming potential (GWP) for the warming OTCs and the control plots during growing seasons from 2011 to 2013 in Changbai Mountain Tundra.

	Control plots	Warming chambers	*P* value
2011			
CO_2_ flux (mg CO_2_-C m^−2^ h^−1^)	60.1 ± 19.3	144.9 ± 25.9	0.035
CH_4_flux (μg CH_4_-C m^−2^ h^−1^)	−9.7 ± 19.4	−21.5 ± 10.0	0.042
N_2_O flux (μg N_2_O-N m^−2^ h^−1^)	0.43 ± 2.17	0.28 ± 1.87	0.884
Global warming potential	220.0 ± 40.4	530.4 ± 57.7	0.035
2012			
CO_2_ flux (mg CO_2_-C m^−2^ h^−1^)	81.8 ± 8.5	117.6 ± 12.7	0.107
CH_4_flux (μg CH_4_-C m^−2^ h^−1^)	−23.7 ± 3.4	−31.3 ± 4.3	0.121
N_2_O flux (μg N_2_O-N m^−2^ h^−1^)	1.07 ± 0.92	0.15 ± 1.32	0.525
Global warming potential	299.3 ± 20.5	430.5 ± 31.7	0.108
2013			
CO_2_ flux (mg CO_2_-C m^−2^ h^−1^)	82.1 ± 21.1	119.3 ± 24.4	0.024
CH_4_flux (μg CH_4_-C m^−2^ h^−1^)	−14.0 ± 6.1	−27.5 ± 4.1	0.024
N_2_O flux (μg N_2_O-N m^−2^ h^−1^)	−0.08 ± 0.87	−1.95 ± 0.73	0.042
Global warming potential	300.4 ± 45.4	436.8 ± 53.9	0.025
